# First Report of Anuran *Trypanosoma* DNA in Flat-Tailed House Geckos (Reptilia: Gekkonidae) Collected from Southern Thailand: No Evidence as a Reservoir for Human Trypanosomatids

**DOI:** 10.3390/pathogens11020247

**Published:** 2022-02-14

**Authors:** Prapimporn Toontong, Sakone Sunantaraporn, Sonthaya Tiawsirisup, Theerakamol Pengsakul, Rungfar Boonserm, Atchara Phumee, Padet Siriyasatien, Kanok Preativatanyou

**Affiliations:** 1 Medical Parasitology Program, Department of Parasitology, Faculty of Medicine, Chulalongkorn University, Bangkok 10330, Thailand; prapimpornfang@gmail.com; 2 Medical Science Program, Faculty of Medicine, Chulalongkorn University, Bangkok 10330, Thailand; narmspace_open@hotmail.com; 3 Vector Biology and Vector-Borne Disease Research Unit, Department of Parasitology, Faculty of Medicine, Chulalongkorn University, Bangkok 10330, Thailand; sky_rung123@hotmail.com (R.B.); padet.s@chula.ac.th (P.S.); 4 Animal Vector-Borne Disease Research Unit, Department of Veterinary Pathology, Faculty of Veterinary Science, Chulalongkorn University, Bangkok 10330, Thailand; sonthaya.t@chula.ac.th; 5 Faculty of Medical Technology, Prince of Songkla University, Songkhla 90110, Thailand; theerakamol.p@psu.ac.th; 6 Department of Medical Technology, School of Allied Health Sciences, Walailak University, Nakhon Si Thammarat 80610, Thailand; atchara.ph@wu.ac.th; 7 Excellent Center for Dengue and Community Public Health (EC for DACH), Walailak University, Nakhon Si Thammarat 80610, Thailand

**Keywords:** *Trypanosoma*, *Leishmania*, flat-tailed house gecko, sand flies, *SSU rRNA* gene, *Cytb* gene

## Abstract

Over the years, cases of autochthonous leishmaniasis have been dramatically increasing in Thailand. Recently, several publications have claimed certain species of the phlebotomine sand flies and biting midges potentially serve as natural vectors of *Leishmania* and *Trypanosoma* species in this country. However, more information regarding the vector–parasite relationships, as well as their natural reservoirs in the country, still needs to be explored. Herein, we hypothesized that synanthropic reptiles in the leishmaniasis-affected area might be a natural reservoir for these parasites. In this present study, a total of nineteen flat-tailed house geckos were collected from the house of a leishmaniasis patient in Songkhla province, southern Thailand, and then dissected for their visceral organs for parasite detection. Small subunit ribosomal RNA (*SSU rRNA*) gene and internal transcribed spacer 1 (*ITS-1*)-specific amplifications were conducted to verify the presence of *Trypanosoma* and *Leishmania* parasites, respectively. Only *Trypanosoma* DNA was screened positive in eight gecko individuals by *SSU rRNA*-PCR in at least one visceral organ (4, 4, and 6 of the heart, liver, and spleen, respectively) and phylogenetically related to the anuran *Trypanosoma* spp. (An04/Frog1 clade) previously detected in three Asian sand fly species (*Phlebotomus kazeruni*, *Sergentomyia indica*, and *Se. khawi*). Hence, our data indicate the first detection of anuran *Trypanosoma* sp. in the flat-tailed house geckos from southern Thailand. Essentially, it can be inferred that there is no evidence for the flat-tailed house gecko (*Hemidactylus platyurus*) as a natural reservoir of human pathogenic trypanosomatids in the leishmaniasis-affected area of southern Thailand.

## 1. Introduction

*Trypanosoma* and *Leishmania* are kinetoplastid flagellate parasites belonging to the family Trypanosomatidae. These parasites can infect a wide range of hosts, including humans, and domestic and wild animals, and have a substantial impact on public health worldwide [1,2]. In endemic areas, *Trypanosoma* causes two important human infectious diseases, including American trypanosomiasis (Chagas disease), caused by *T. cruzi*, and African trypanosomiasis (sleeping sickness), caused by *T. brucei* (*T. b. rhodesiense* and *T.b. gambiense*) [3]. Human leishmaniasis, caused by *Leishmania*, can present three multi-spectral forms of clinical manifestations, i.e., visceral, cutaneous, and mucocutaneous leishmaniases [4]. *Trypanosoma* is transmitted by a range of hematophagous insects, including triatomine bugs, tsetse flies, mosquitoes, sand flies, and blood-sucking leeches [5]. Phlebotomine sand flies are principally natural vectors responsible for leishmaniasis [4]. Recently, certain species of biting midges, *Culicoides* Latreille, have also, for the first time, been reported to be the potential vectors of *L. martiniquensis* and *Trypanosoma* sp. in Thailand [6]. 

In Thailand, the number of autochthonous leishmaniasis cases is continuously increasing, mostly in the northern and southern provinces. To date, over 20 indigenous leishmaniasis cases have been reported in the country since 1996. Accordingly, most of these autochthonous cases have been diagnosed for *L. martiniquensis* and *L. orientalis* [7,8,9]. Traditionally, trypanosomiasis in non-endemic areas is commonly prevalent in a broad range of domestic and wild animals, i.e., rodents, cattle, buffalos, elephants, tigers, and pigs, as well as amphibians and reptiles [1,10]. However, a number of atypical human trypanosomiasis cases have so far been documented worldwide and ascribed to some animal trypanosomes, such as *T. b. brucei, T. evansi*, *T. vivax*, *T. congolense*, *T. lewisi*, and *T. lewisi*-like trypanosomes [11,12,13]. Of interest, atypical trypanosomiasis caused by *T. lewisi*-like infection has been reported in an infant in northern Thailand [13]. Therefore, the increasing cases of autochthonous leishmaniasis, as well as emerging atypical trypanosomiasis, have raised the interesting question of whether there are natural reservoirs for trypanosomatid parasites, especially in affected areas of Thailand. 

Reptiles have been proven to be reservoirs of several pathogens, including protozoa, and helminths, as well as arthropod ectoparasites [14]. Relevant to human trypanosomatids, *T. brucei* was isolated from monitor lizards (*Varanus niloticus*) in the sleeping sickness-endemic area [15,16]. In addition, the DNA of reptilian *Leishmania* (*Sauroleishmania*) and mammalian *Leishmania* species (i.e., *L. donovani*, *L. tropica*, and *L. turanica*) has been recently detected in some desert lizards and snakes from the endemic areas in northwest China [17,18]. It has also been found that *L. tropica* from lizards share common ancestry with those detected from clinical cases, suggesting their potential role as a natural reservoir [17]. Therefore, we hypothesized that other synanthropic reptiles, especially house geckos, in the affected area in Thailand may potentially act as a natural source of infection of trypanosomatids pathogenic to humans.

In this study, we aimed to survey the presence of *Trypanosoma* and *Leishmania* parasites in flat-tailed house geckos collected from the house of a leishmaniasis patient in Songkhla province, southern Thailand. The findings from this study might help us better understand the transmission of *Trypanosoma* and *Leishmania* parasites. Furthermore, our study would be useful for future studies for the effective prevention and control of emerging diseases caused by trypanosomatid parasites in Thailand.

## 2. Results

### 2.1. Molecular Detection of Trypanosoma and Leishmania DNA from Dissected Organ Specimens

From 19 flat-tailed house geckos, 57 dissected organs consisting of the heart, liver, and spleen of each individual were extracted for genomic DNA. Then, *SSU rRNA* and *ITS1*-specific amplification were conducted to detect *Trypanosoma* and *Leishmania*, respectively. The results showed that eight flat-tailed house geckos tested positive for *Trypanosoma* DNA in at least one organ, as detailed in Table 1. However, *Leishmania* DNA was not detected in any specimen. Of these, only one flat-tailed house gecko (J4) tested positive for *Trypanosoma* DNA in all dissected organs. 

### 2.2. Nucleotide BLAST Analysis and Phylogenetic Tree Construction 

The nucleotide sequences of partial *SSU rRNA* genes obtained from fourteen positive organs in the PCR step harbored a size ranging from approximately 948 to 956 bp. The BLASTn results revealed that all fourteen partial *SSU rRNA* sequences in this study showed the highest similarity to *Trypanosoma SSU rRNA* reference derived from *Sergentomyia indica* (accession no. MK603820) with a percentage of identity (98.25–100%) and significant E-Value. Then, all these sequences were annotated and submitted to the GenBank for assigning accession numbers as MN629898–MN629905 and MN629907–MN629912. Additionally, a multiple sequence alignment was performed by using ClustalW implemented in the BioEdit Sequence Alignment Editor Version 7.2.6, showing pairwise intraspecific genetic variability ranging 0–4.2% as demonstrated in Table S1.

**Figure 1:**
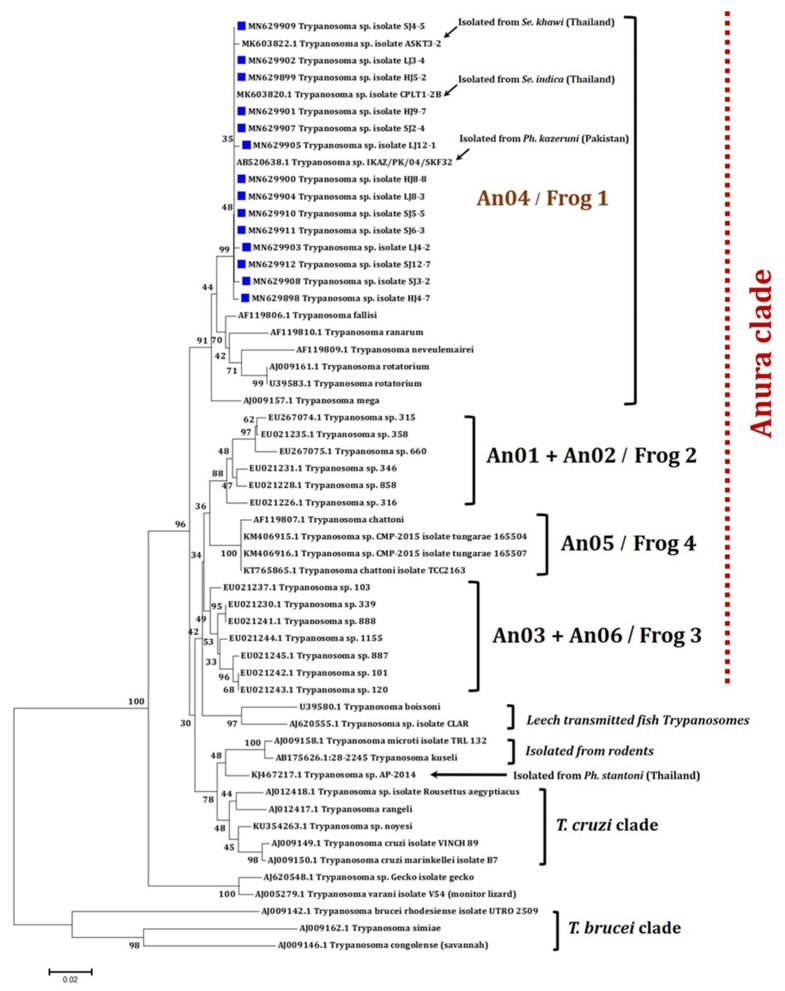
Maximum likelihood (ML) tree of 55 enrolled *SSU rRNA* sequences of *Trypanosoma* spp. constructed by the K2P+G+I model with 1000 bootstrap tests. The *Trypanosoma* sp. sequences obtained from this study are indicated with blue squares.

Fifty-five partial *Trypanosoma SSU rRNA* sequences consisting of fourteen sequences in this study and other forty-one sequences available in the GenBank database were enrolled for the phylogenetic construction as illustrated in Figure 1. Consistent with the BLASTn results, it was demonstrated that all newly obtained *Trypanosoma* sequences were almost identical and closely related with *Trypanosoma* sequences formerly detected in three Asian sand fly species: *Se. indica* (MK603820) and *Se. khawi* (MK603822) from Thailand, and *Phlebotomus kazeruni* (AB520638) from Pakistan. Moreover, our *Trypanosoma* sp. sequences were distinctively clustered in the main clade of an An04/Frog1 group, as depicted in Figure 1, clearly inferring that the *Trypanosoma* sp. sequences detected in this study were classified as the amphibian *Trypanosoma* species.

**Figure 2:**
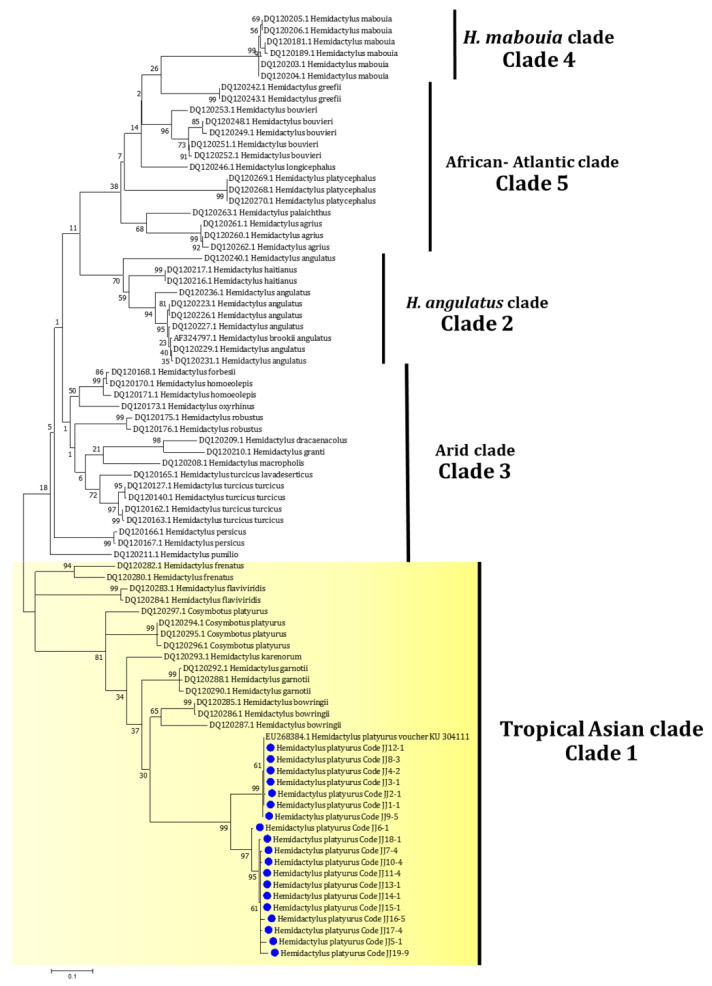
Maximum likelihood (ML) tree of 83 enrolled partial mitochondrial *Cytb* sequences of the house geckos based on the HKY+G+I model of nucleotide substitution with 1000 bootstrap tests. Blue circles represent the sequences of flat-tailed house geckos in this study.

For gecko species identification, mitochondrial *Cytb* gene-specific PCR yielded a band of approximately 376 bp and the BLASTn result revealed that all collected samples were *Hemidactylus platyurus* with significant E-Value. Concordantly, a maximum likelihood tree, including the total of 83 *Cytb* sequences from the *Hemidactylus* geckos in this study and those from the GenBank database, was constructed, showing that all our sequences were clustered closely with *H. platyurus* (EU268384) in the tropical Asian clade, as demonstrated in Figure 2. The partial *Cytb* sequences were submitted to the GenBank and assigned the accession numbers as MN635549–MN635567.

## 3. Discussion

Trypanosomatids, including *Trypanosoma* and *Leishmania*, are parasites causing vector-borne neglected infectious diseases in both humans and animals worldwide. Basically, trypanosomiasis can be transmitted to humans and other mammals by infective metacyclic trypomastigotes mainly via the bite or contact with feces or urine of arthropod vectors [19,20]. For leishmaniasis, the transmission occurs via the bite of infected female phlebotomine sand flies [7]. In addition, several publications demonstrated that a variety of animals can serve as potential reservoirs of both *Trypanosoma* [21,22,23] and *Leishmania* [24,25,26]. Therefore, the investigation of animal reservoirs would contribute to a better understanding of the biology of pathogens and the effective control of disease transmission. However, there are still limitations in the data concerning animal reservoirs of the human pathogenic trypanosomatid parasites in Thailand.

Since 2014, we have visited the house of an HIV-infected male patient to collect sand flies. He was diagnosed and completely treated for his indigenous leishmaniasis seven years ago [27,28]. Although this patient has been entirely cured and is not an active case now, our recent investigation reveals that sand flies collected from his house area still screened positive for DNA of *L. martiniquensis* and *Trypanosoma* sp. [29,30]. Additionally, we observed that several flat-tailed house geckos could be easily found on his house wall; therefore, it is reasonable for us to hypothesize that the flat-tailed house gecko might play a role as a reservoir for *Trypanosoma* and/or *Leishmania* parasites. Thus, *SSU rRNA* and *ITS1*-specific PCR were designed to display the presence of *Trypanosoma* and *Leishmania* DNA in the gecko visceral organs. As a result, only *Trypanosoma* DNA was found in eight geckos with at least one visceral organ by *SSU rRNA*-based PCR. 

Intriguingly, *Trypanosoma* sp. DNA could be detected in the six spleen samples from eight positive geckos, suggesting that the parasites preferentially deposited in particular tissues. Consistently, several *Trypanosoma* species, including *T. brucei*, *T. cruzi*, *T. vivax*, and *T. congolense,* have also been mentioned for tissue tropism, especially in extravascular tissues, i.e., the brain, aqueous humor, heart, lung, liver, and kidney, as well as the spleen [31,32,33,34]. This phenomenon would be beneficial to parasites in terms of enhancing transmission, virulence reduction, immune evasion, and resistance to treatment [31]. Unfortunately, blood smear preparation for the microscopic detection of parasites was not conducted in this study due to the inadequate blood volume of the geckos during dissected organ preparations. 

All *Trypanosoma* sp. sequences amplified from positive organs in this study phylogenetically resembled other *Trypanosoma* spp. sequences detected in sand flies from leishmaniasis-endemic areas as follows: *Ph*. *kazeruni* (AB520638) from Pakistan [35], and *Se. khawi* (MK603822) and *Se. indica* (MK603820), as previously described in southern Thailand [30]. This finding suggests the possible transmission of parasites from sand flies to flat-tailed house geckos. In addition, these *Trypanosoma* sequences were grouped into a single clade (An04/Frog1) of previously described anuran or amphibian *Trypanosoma* species, including *T. fallisi*, *T. ranarum*, *T. neveulemairei*, *T. rotatorium*, and *T. mega*, obviously separating from *Trypanosoma* spp. In other clades [36]. As these are positioned in the An04/Frog1 clade, this implies that *Trypanosoma* sp. identified in flat-tailed house geckos might be able to infect different kinds of hosts, including both amphibians and reptiles. 

Based on *ITS1*-PCR in the present study, we could not detect both reptilian and mammalian *Leishmania* DNA in any gecko organ samples. However, our previous publication revealed that *L. martiniquensis* DNA was detected in the liver of black rats (*Rattus rattus*) collected from the house area of the same patient in this study by *ITS1*-PCR [37]. Furthermore, *L. martiniquensis* has been reported in horses in Central Europe and the USA, as well as bovines in Switzerland [38,39,40]. Recently, the DNA of *L. martiniquensis* was detected in the buffy coat of a black rat captured from Chiang Rai province, northern Thailand [41]. To our knowledge, it can be presumed that mammalian vertebrate hosts most likely serve as a natural reservoir and involve the zoonotic transmission of *L. martiniquensis* in Thailand.

In addition to *Leishmania*, phlebotomine sand flies have been also mentioned to serve as the potential vectors of *Trypanosoma* species causing trypanosomiasis in a wide variety of vertebrate hosts, including fishes, frogs, lizards, birds, and mammals [35,42,43,44,45]. Previous publications have demonstrated that *Leishmania* and *Trypanosoma* DNA could be detected in phlebotomine sand flies, especially in the affected areas of Thailand [29,30,37,46,47]. *L. martiniquensis* DNA could be detected in several sand fly species, i.e., *Se. khawi*, *Se. barraudi* [30,37,46], and *Se. iyengari* [47]. For *Trypanosoma*, the first detection was described in *Ph. stantoni* by Phumee et al. (2017) [29]. Afterward, *Trypanosoma* sp. DNA was detected in *Se. khawi*, *Se. indica*, *Se. anodontis*, *Ph. asperulus*, and *Ph. betisi* by Srisuton et al. (2019) [30]. Interestingly, *L. martiniquensis* and *Trypanosoma* sp. co-infection was found in one *Se. khawi* sample collected near the house of a leishmaniasis patient in Songkhla province of Thailand, inferring that these two related trypanosomatids could harbor the same sand fly vector and consequently share the same animal host upon which such vectors feed [30]. By analyzing blood meal, Siripattanapipong et al. (2018) revealed that the blood DNA of both humans and the sun skink (*Mabuya multifasciata*), taxonomically related to the house gecko, could be detected in *Se. iyengari* sand flies [47]. Altogether with our results, we speculated that sand flies could feed on and transmit the pathogens to flat-tailed house geckos, serving as a natural host of anuran *Trypanosoma* parasites. 

For the feasibility of anuran *Trypanosoma* to infect mammalian hosts, Hysek and Zizka (1976) demonstrated experimental transmission and pathogenic effects of *T. rotatorium* in white laboratory mice [48]. Contrastingly, Kato et al. (2010) inoculated intradermally with anuran *Trypanosoma* sp. (code: IKAZ/PK/04/SKF32), isolated from the sand fly *Ph. kazeruni* in Pakistan, into Mongolian gerbils and BALB/c mice; however, parasite DNA could not be detected in both peripheral blood and lymphatic tissues by PCR 6 weeks after infection [35]. Interestingly, the *SSU rRNA* sequence (AB520638) of this anuran *Trypanosoma* sp. (code: IKAZ/PK/04/SKF32) was closely clustered with our *Trypanosoma* sp. Sequences, as revealed in Figure 1. This strongly suggests that the *Trypanosoma* sp. detected in flat-tailed house geckos in this study likely possesses a high degree of specificity, restrictive to amphibian or reptilian hosts. In addition, only certain species of animal trypanosomes, i.e., *T. b. brucei*, *T. evansi*, *T. vivax*, *T. congolense*, *T. lewisi*, and *T. lewisi*-like trypanosomes, have so far been documented for nineteen cases of atypical human trypanosomiasis worldwide over 1917–2010, as reviewed by Truc et al. (2013) [11]. To the best of our knowledge, there are no published data now indicating that anuran *Trypanosoma* species can cause atypical infections or any diseases in humans.

Essentially, it can be inferred from our molecular findings that the flat-tailed house gecko can be a natural host of anuran *Trypanosoma* species, and therefore, there is no promising evidence that it acts as a reservoir for trypanosomatid parasites relevant to human health. Future studies should focus on other domestic animals (e.g., cattle, dogs, and cats) to see whether they could be a natural reservoir host of human pathogenic trypanosomatids, especially in the affected area. Ultimately, a deep understanding of the relationships of parasites, vectors, and reservoirs could be used to deploy the effective prevention and control of transmission of both *Trypanosoma* and *Leishmania* parasites in Thailand.

## 4. Materials and Methods

### 4.1. Ethics Statement 

The study on animals was approved by the animal research ethics committee of Chulalongkorn University Animal Care and Use Protocol (CU-ACUP), Faculty of Medicine, Chulalongkorn University, Bangkok, Thailand (COA No. 004/2562).

### 4.2. Gecko Collection and Visceral Organ Dissection

**Figure 3:**
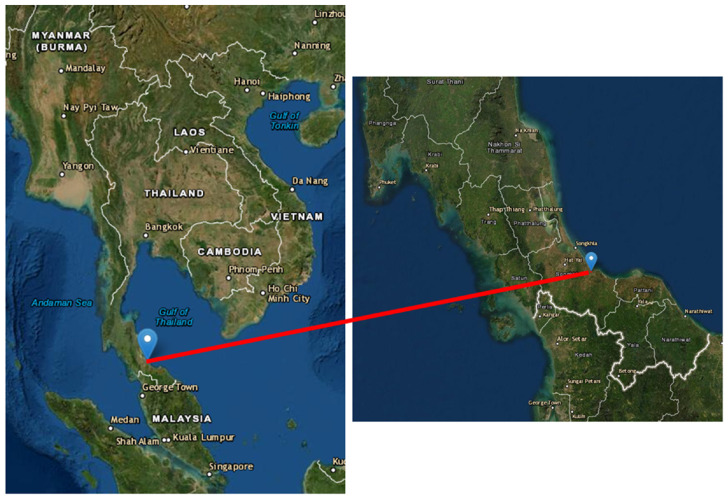
Map of Thailand, showing the location of the house of a leishmaniasis patient for flat-tailed house gecko collection in Na Thawi district (6°44’30” N, 100°41’30” E), Songkhla province, southern Thailand. The map photograph was adapted from the free public domain (https://earthexplorer.usgs.gov/; accessed on 1 November 2021).

A total of 19 flat-tailed house geckos were captured alive for a duration of one week from the house of a 56-year-old HIV-infected male Thai rubber planter, who was first reported in July 2012 with a history of being diagnosed with autochthonous cutaneous, visceral leishmaniasis caused by *L. martiniquensis*, at Na Thawi district (6°44’30” N, 100°41’30” E), Songkhla province, southern Thailand, as shown in Figure 3 [27]. The collected flat-tailed house geckos were anesthetized by Zoletil^®^ 100 (Virbac, Carros, France). Then, samples of liver, heart, and spleen were dissected from individuals, placed in sterile 1× phosphate buffer saline, and stored at 4 °C. All samples were taken to the Vector Biology and Vector-Borne Disease Research Unit, Department of Parasitology, Faculty of Medicine, Chulalongkorn University for further DNA extraction and parasite detection.

### 4.3. Genomic DNA Extraction

Total DNA was isolated from fifty-seven dissected preparations of the liver, heart, and spleen (three organs for each sample) using the Invisorb^®^ Spin Tissue mini kit (STRATEC, Birkenfeld, Germany) according to the manufacturer’s instructions. Each sample was lysed in a 400 µL of lysis buffer G with 40 µL of proteinase K. Purified genomic DNA was eluted in 50 µL of elution buffer. Then, DNA concentrations of extracted samples were measured by the Nanodrop 2000c spectrophotometer (Thermo Fisher Scientific, Waltham, MA, USA). The purified genomic DNA was used as a DNA template in a PCR step and kept at −20 °C for long-term storage.

### 4.4. Detection of Trypanosoma and Leishmania DNA in Flat-Tailed House Geckos

The purified genomic DNA was used to detect *Trypanosoma* and *Leishmania* parasites by using conventional PCR. To detect *Trypanosoma*, a set of primers (TRY927-F: 5´-GAA-ACA-AGA-AAC-ACG-GGA-G-3´ and TRY927-R: 5´-CTA-CTG-GGC-AGC-TTG-GA-3´) were applied to amplify approximately 900 bp product of the *SSU rRNA* gene as described by Noyes et al. (1999) [49]. PCR reagents were set up in a total volume of 25 µL, containing 50 ng of genomic DNA, 10× PCR buffer, 25 mM of MgCl_2_ (Thermo Fisher Scientific, Waltham, MA, USA), 2.5 mM of dNTPs (GeneAll, Seoul, Korea), 10 µM of forward and reverse primers, and 1 unit of *Taq* DNA polymerase (Thermo Fisher Scientific, Waltham, MA, USA). The PCR condition was programmed by pre-denaturation at 95 °C for 5 min, followed by 40 cycles of denaturation at 95 °C for 45 s, annealing at 53 °C for 1 min, and extension at 72 °C for 1.30 min. The final extension temperature was at 72 °C and extended for 7 min. 

To detect *Leishmania* DNA, a pair of primers (LeF: 5´-TCC-GCC-CGA-AAG-TTC-ACC-GAT-A-3´ and LeR: 5´-CCA-AGT-CAT-CCA-TCG-CGA-CAC-G-3´) were used as previously described by Spanakos et al. (2008) to amplify the approximately 379 bp fragment of internal transcribed spacer 1 (*ITS1*) region [50]. The PCR amplification profile includes pre-denaturation at 95 °C for 5 min, followed by 40 cycles of denaturation at 95 °C for 1 min, annealing at 65 °C for 1 min, extension at 72 °C for 1 min, and final extension at 72 °C for 7 min. The PCR reagents were identical to those used for *Trypanosoma* detection as described above. The PCR amplicons were separated on a 1.5% (*w*/*v*) agarose gel electrophoresis and visualized with Quantity One Quantification Analysis Software Version 4.5.2 (Gel Doc EQ System; Bio-Rad, Hercules, CA, USA) after being stained with ethidium bromide. In this study, plasmid DNA containing *SSU rRNA* and *ITS1* genes were used as the positive controls in *Trypanosoma* and *Leishmania* detection, respectively, whereas double-distilled water (ddH_2_O) was used as a negative control.

### 4.5. Molecular Identification of Flat-Tailed House Geckos

To identify the species of geckos, the partial mitochondrial cytochrome b (*Cytb*) gene was amplified using oligonucleotide primers previously described as follows: CytbF: 5´-AAA-AAG-CTT-CCA-TCC-AAC-ATC-TCA-GCA-TGA-TGA-AA-3´ and CytbR: 5´-AAA-CTG-CAG-CCC-CTC-AGA-ATG-ATA-TTT-GTC-CTC-A-3´ [51,52]. Using 50 ng of DNA template and the same reagents as detailed above, PCR was performed under the following thermal profiles: 95 °C for 5 min, 35 cycles of denaturation at 95 °C for 30 s, annealing at 45 °C for 45 s, and extension at 72 °C for 1 min, followed by a final extension at 72 °C for 7 min. Amplification of the mitochondrial *Cytb* gene was determined by the presence of a band at approximately 376 bp.

### 4.6. TA Cloning and Sanger Sequencing

All positive PCR products were inserted into pGEM^®^ T-Easy vector (Promega Corporation, Madison, WI, USA), following the manufacturer’s instructions. Recombinant plasmids were chemically transformed into competent *Escherichia coli* strain DH5α cells and then screened by the blue-white colonies selection system on the Ampicillin/X-Gal/IPTG Luria Bertani agar plate. Positive white clones were ensured by colony PCR and subsequently cultured overnight in LB medium with ampicillin. Plasmids with inserts were extracted using the Invisorb^®^ Spin Plasmid Mini Kit (STRATEC, Birkenfeld, Germany) and sent to the Macrogen, Inc. (Seoul, Korea) for Sanger sequencing service. 

### 4.7. Phylogenetic Tree Construction 

Retrieved sequence reads were analyzed using the BioEdit Sequence Alignment Editor Version 7.2.6 for removal of plasmid vector sequences [53]. Trimmed sequences were then compared to the GenBank reference database using the BLASTn search tool (https://blast.ncbi.nlm.nih.gov/Blast.cgi, accessed on 15 November 2021) for species identification. All *SSU rRNA* and *Cytb* nucleotide sequences obtained from this study were submitted to the GenBank database and assigned accession numbers, as detailed in the results. 

For phylogenetic analysis, a tree of *SSU rRNA Trypanosoma* sequences in this study and from the GenBank database was built by the MEGAX software using the maximum-likelihood method with Kimura-2 parameter (K2P) with invariant positions and gamma distribution (K2P+G+I) and tested by 1000 bootstrap replicates [54]. Likewise, phylogeny based on the *Cytb* sequences of the house geckos in this study and those from the GenBank database was analyzed by the maximum likelihood method using the Hasegawa-Kishino-Yano model with invariant positions and gamma distribution (HKY+G+I) with 1000 bootstrap tests. 

## 5. Conclusions

The natural infection by the trypanosomatid parasites in flat-tailed house geckos (*H. platyurus*) was surveyed in the affected area of leishmaniasis in Songkhla, southern Thailand. Based on the preliminary data in this study, it can be inferred that the flat-tailed house gecko can serve as a natural host of amphibian *Trypanosoma* species, phylogenetically clustered in the anuran An04/Frog1 clade, and therefore has no significant role in the transmission of human pathogenic trypanosomatids. Conclusively, the detection and identification of the trypanosomatid parasites in their natural hosts would help us understand the host-parasite relationships as well as the biology and dynamics of parasite transmission better. 

## Figures and Tables

**Table 1:** *SSU rRNA*-based detection of *Trypanosoma* DNA in visceral organs dissected from nineteen flat-tailed house geckos and species identification based on mitochondrial *Cytb* sequences.

Isolate Code	*SSU rRNA*-PCR			Gecko Species Identification
	Heart (H)	Liver (L)	Spleen (S)	
J1	−	−	−	*Hemidactylus platyurus*
J2	−	−	+	*Hemidactylus platyurus*
J3	−	+	+	*Hemidactylus platyurus*
J4	+	+	+	*Hemidactylus platyurus*
J5	+	−	+	*Hemidactylus platyurus*
J6	−	−	+	*Hemidactylus platyurus*
J7	−	−	−	*Hemidactylus platyurus*
J8	+	+	−	*Hemidactylus platyurus*
J9	+	−	−	*Hemidactylus platyurus*
J10	−	−	−	*Hemidactylus platyurus*
J11	−	−	−	*Hemidactylus platyurus*
J12	−	+	+	*Hemidactylus platyurus*
J13	−	−	−	*Hemidactylus platyurus*
J14	−	−	−	*Hemidactylus platyurus*
J15	−	−	−	*Hemidactylus platyurus*
J16	−	−	−	*Hemidactylus platyurus*
J17	−	−	−	*Hemidactylus platyurus*
J18	−	−	−	*Hemidactylus platyurus*
J19	−	−	−	*Hemidactylus platyurus*
Total	4	4	6	
		14		

‘−’, not detected; ‘+’, detected.

## Data Availability

Not applicable.

## Supplementary Materials

The following supporting information can be downloaded at: https://www.mdpi.com/article/10.3390/pathogens11020247/s1, Table S1: Identity matrix showing the percentage of partial *SSU rRNA* sequence similarity between PCR-positive isolates.
