# Supplementary material for: First Report of Anuran Trypanosoma DNA in Flat-Tailed House Geckos (Reptilia: Gekkonidae) Collected from Southern Thailand: No Evidence as a Reservoir for Human Trypanosomatids
Source: Pathogens. 2022 Feb 14;11(2):247. doi: 10.3390/pathogens11020247 (PMC8877104; doi:10.3390/pathogens11020247)
Supplement: Supplementary file 1 [file pathogens-11-00247-s001.zip › pathogens-1579649-supplementary.pdf]

Table S1. Identity matrix showing the percentage of partial *SSU rRNA* sequence similarity between PCR-positive isolates listed in Table 1. The first alphabet codes H, L, and S represent the heart, liver, and spleen, respectively.

Sequence	SJ2	LJ3	SJ3	HJ4	LJ4	SJ4	HJ5	SJ5	SJ6	HJ8	LJ8	HJ9	LJ12	SJ12
SJ2														
LJ3	99.7													
SJ3	99.2	99.4												
HJ4	98.4	98.3	97.7											
LJ4	99.4	99.2	98.7	97.9										
SJ4	99.8	99.6	99.1	98.3	99.3									
HJ5	99.7	100	99.4	98.3	99.2	99.6								
SJ5	100	99.7	99.2	98.4	99.4	99.8	99.7							
SJ6	98.9	99.1	98.6	97.4	98.4	98.8	99.1	98.9						
HJ8	100	99.7	99.2	98.4	99.4	99.8	99.7	100	98.9					
LJ8	99.4	99.2	98.7	97.9	98.9	99.3	99.2	99.4	98.4	99.4				
HJ9	100	99.7	99.2	98.4	99.4	99.8	99.7	100	98.9	100	99.4			
LJ12	98.9	99.0	98.5	97.4	98.4	98.8	99.0	98.9	98.3	98.9	98.7	98.9		
SJ12	96.5	96.8	96.2	95.8	96.0	96.4	96.8	96.5	96.9	96.5	96.3	96.5	96.9	
